# 19233 Basis profile curve identification to understand electrical stimulation effects in human brain networks

**DOI:** 10.1017/cts.2021.423

**Published:** 2021-03-30

**Authors:** Kai J. Miller, Klaus-Robert Muller, Dora Hermes

**Affiliations:** 1 Mayo Clinic; 2 Google Brain, Berlin

## Abstract

ABSTRACT IMPACT: Brain networks can be explored by delivering brief pulses of electrical current in one area while measuring responses in other areas, and this describes an open-source novel algorithm to carry out this exploration. OBJECTIVES/GOALS: If we focus on a single brain site and observe the average effect of stimulating each of many other brain sites, visually-apparent motifs in the temporal response shape emerge from adjacent stimulation sites. There are no existing approaches to identify and quantify the spatiotemporal structure of these motifs. METHODS/STUDY POPULATION: Individual stimulation trials are correlated with one another, then a correlation-significance matrix quantifying similarity between stimulation sites is decomposed with non-negative matrix factorization, in which the inner dimension is iteratively reduced. The dimensionality reduction identifies stimulation sites that produce a common elicited temporal response, and linear kernel PCA is applied to obtain the robust profile of this response cluster. RESULTS/ANTICIPATED RESULTS: We describe and illustrate a data-driven approach to determine characteristic spatiotemporal structure in these response shapes, summarized by a set of unique ‘basis profile curves’ (BPCs). Each BPC may be mapped back to underlying anatomy in a natural way, quantifying projection strength from each stimulation site using simple metrics. Our technique is demonstrated for an array of implanted brain surface electrodes in a human patient, and our code is shared at https://purl.stanford.edu/rc201dv0636. DISCUSSION/SIGNIFICANCE OF FINDINGS: This framework enables straightforward interpretation of single-pulse brain stimulation data, and can be applied generically to explore the diverse milieu of interactions that comprise the connectome.

